# Cost-Effectiveness of Adjuvant Immunotherapy With Cytokine-Induced Killer Cell for Hepatocellular Carcinoma Based on a Randomized Controlled Trial and Real-World Data

**DOI:** 10.3389/fonc.2021.728740

**Published:** 2021-12-03

**Authors:** Jeong-Yeon Cho, Sun-Hong Kwon, Eui-Kyung Lee, Jeong-Hoon Lee, Hye-Lin Kim

**Affiliations:** ^1^ School of Pharmacy, Sungkyunkwan University, Suwon, South Korea; ^2^ Department of Internal Medicine and Liver Research Institute, Seoul National University College of Medicine, Seoul, South Korea; ^3^ College of Pharmacy, Sahmyook University, Seoul, South Korea

**Keywords:** cost-effectiveness, immunotherapy, adjuvant therapy, cytokine-induced killer cell, economic evaluation, hepatocellular carcinoma

## Abstract

**Background:**

Studies using data from randomized controlled trials (RCTs) and real-world data (RWD) have suggested that adjuvant cytokine-induced killer (CIK) cell immunotherapy after curative treatment for hepatocellular carcinoma (HCC) prolongs recurrence-free survival (RFS) and overall survival (OS). However, the cost-effectiveness of CIK cell immunotherapy as an adjuvant therapy for HCC compared to no adjuvant therapy is uncertain.

**Methods:**

We constructed a partitioned survival model to compare the expected costs, life-year (LY), and quality-adjusted life-year (QALY) of a hypothetical population of 10,000 patients between CIK cell immunotherapy and no adjuvant therapy groups. Patients with HCC aged 55 years who underwent a potentially curative treatment were simulated with the model over a 20-year time horizon, from a healthcare system perspective. To model the effectiveness, we used OS and RFS data from RCTs and RWD. We estimated the incremental cost-effectiveness ratios (ICERs) and performed extensive sensitivity analyses.

**Results:**

Based on the RCT data, the CIK cell immunotherapy incrementally incurred a cost of $61,813, 2.07 LYs, and 1.87 QALYs per patient compared to no adjuvant therapy, and the estimated ICER was $33,077/QALY. Being less than the willingness-to-pay threshold of $50,000/QALY, CIK cell immunotherapy was cost-effective. Using the RWD, the ICER was estimated as $25,107/QALY, which is lower than that obtained using RCT. The time horizon and cost of productivity loss were the most influential factors on the ICER.

**Conclusion:**

We showed that receiving adjuvant CIK cell immunotherapy was more cost-effective than no adjuvant therapy in patients with HCC who underwent a potentially curative treatment, attributed to prolonged survival, reduced recurrence of HCC, and better prognosis of recurrence. Receiving CIK cell immunotherapy may be more cost-effective in real-world clinical practice.

## Introduction

Hepatocellular carcinoma (HCC) is an aggressive and frequently occurring cancer, with approximately 670,000 new cases and 625,000 deaths worldwide in 2018 (1). Interestingly, most HCC cases occur in individuals with well-known risk factors, such as chronic hepatitis B virus (HBV) or hepatitis C virus (HCV) infection, nonalcoholic steatohepatitis, chronic alcoholism, and liver cirrhosis. Thus, a regular surveillance program for populations with such risk factors is recommended to detect HCC at an early stage, and more than half of new HCC cases are now diagnosed at very early or early stages in Japan and Taiwan owing to the implementation of nationwide surveillance programs (2).

Generally, in the very early or early stages of HCC, potentially curative treatments, such as surgical resection or radiofrequency ablation (RFA), can be applied; however, even after successful resection, 50–70% of patients experience recurrence within 5 years (3, 4). The long-term prognosis of patients with recurrent HCC remains poor because of deterioration of liver function with repeated recurrences even after curative treatment following early detection of HCC and during a successful regular surveillance program (5). Therefore, in the treatment of HCC, recurrence after curative treatment is an important indicator that is negatively related to long-term survival (6). To improve recurrence-free survival (RFS) in patients with HCC, several studies on adjuvant therapy have been conducted; however, none of these studies provide sufficient evidence of improvement except for antiviral treatment for HBV- or HCV-related HCC (3).

Since the first report on the antitumor activity of cytokine-induced killer (CIK) cells, 106 clinical trials for various types of cancers have been registered in the international registry of CIK cells in the past decade (7, 8). Fortunately, recent phase III trials and real-world data (RWD) have shown that adjuvant CIK cell immunotherapy administered after curative treatment for HCC prolongs RFS, cancer-specific survival, and overall survival (OS) with minimum adverse effects. According to previous studies, patients who underwent repeated transfer of individualized autologous CIK cell agents had significantly longer RFS than the control group with a hazard ratio of 0.42 (95% confidence interval [CI], 0.22–0.80) to 0.63 (95% CI, 0.43–0.94) (9–11). These results have generated interest in the economic assessment of the benefits obtained and the input cost for adjuvant CIK immunotherapy. Nevertheless, there is limited data on the cost-effectiveness of immunotherapy in patients with HCC who have undergone potentially curative treatment.

Therefore, it is important for healthcare policymakers, providers, and patients to determine whether adjuvant CIK immunotherapy reflects an appreciable value in the current healthcare environment. We investigated the cost-effectiveness of CIK cell immunotherapy as an adjuvant therapy for patients with HCC using data from a recently reported phase III trial and RWD.

## Materials and Methods

### Overview of Partitioned Survival Model

A cost-utility analysis was performed to evaluate the cost-effectiveness of receiving CIK cell immunotherapy compared with not receiving any adjuvant treatment (no adjuvant therapy) in patients with HCC who underwent a potentially curative treatment (surgical resection, RFA, or percutaneous ethanol injection [PEI]). Aligning with the phase III clinical study (the randomized controlled trial, hereafter referred to as RCT), its extended follow-up study (9, 10), and the phase IV clinical study (hereafter referred to as RWD) of CIK cell immunotherapy (11), we constructed a partitioned survival model, which has been widely used for the economic evaluation of oncology drugs, using Microsoft Excel (Microsoft, Redmond, Washington, USA) (12). The time horizon of the model was twenty years, and the cycle length was three months.

**Figure 1:**
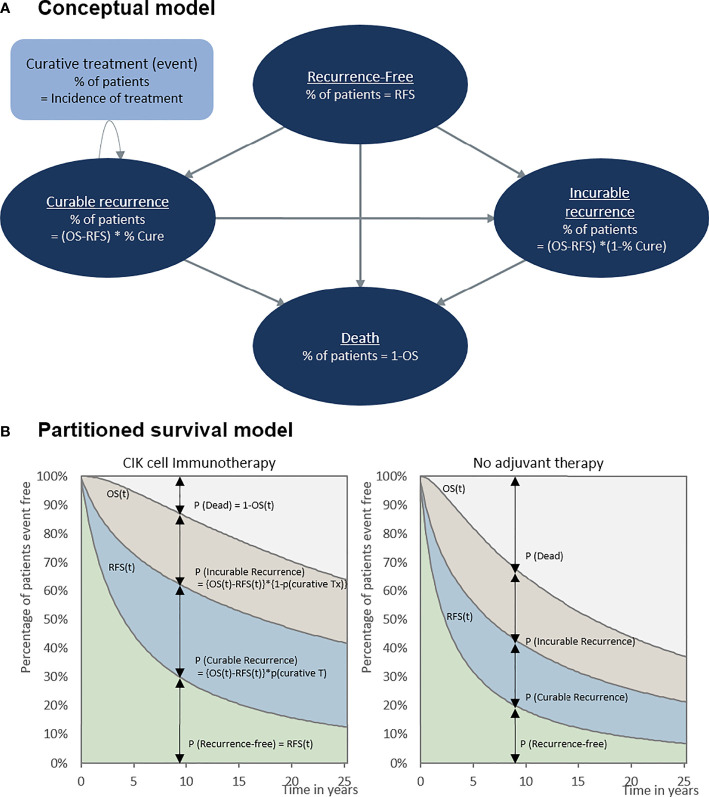
Cost-effectiveness model. **(A)** Conceptual model and **(B)** partitioned survival model. OS, overall survival; RFS, recurrence-free survival.

Our model included four conceptual health states: recurrence-free, curable recurrence, incurable recurrence, and death (**Figure 1A**). Because our population included patients with HCC who underwent a potentially curative treatment, all patients started from the recurrence-free state. RFS was defined as the time from randomization in RCT (in RWD, from curative treatment) to the first recurrence or death. Patients without recurrence remained in the recurrence-free state as the cycle went on. Otherwise, the patient experienced recurrence or death. Unlike a typical partitioned survival model, we divided the recurrence condition into two states according to the curability of treatment to consider the different treatments patients undergo. We assumed that patients received either curative or noncurative treatments once they demonstrated a recurrence. If patients remained in the curable recurrence state, they were treated with curative treatments, such as surgical resection, liver transplantation, RFA, or PEI. By contrast, patients staying in an incurable recurrence state would receive noncurative treatments including transarterial chemoembolization (TACE), external-beam radiation therapy, and systemic therapies including cytotoxic chemotherapy. We performed a *post-hoc* analysis of the records of individual patients on subsequent treatment and follow-up time after recurrences observed in phase III clinical trials to calculate the proportion of patients with curable recurrence and the incidence rates of each curative treatment applied in the curable recurrence state. For the treatment of HCC, liver transplantation, surgical resection, or RFA is considered as potentially curative treatment. In the base-case analysis, according to the international guideline, we assumed that once the patient received other non-curative therapy, such as TACE or systemic therapies including cytotoxic chemotherapy, the recurrence was classified as incurable (13). However, some of these patients might have received TACE for curative intent as curative treatment was not technically feasible; therefore, we adjusted the proportion of curative TACE to the calculation of curable recurrence in the sensitivity analysis. Our model simulated a cohort of 55-year-old patients, considering the mean age of patients in the RCT (9). To validate our model, clinical experts verified the key model assumptions.

The model estimated the costs, gained life-years (LYs), and quality-adjusted life-years (QALYs) for a 20-year time horizon. An annual discount rate of 5% was applied to the outcomes and costs, according to the pharmacoeconomic evaluation guideline from the Health Insurance Review & Assessment Service (HIRA) of the South Korean government (14). This economic evaluation was conducted and reported based on the Consolidated Health Economic Evaluation Reporting Standards (CHEERS) guidelines (15).

### RFS and OS

Two survival curves of RFS and OS derived from the RCTs and RWD were applied in the model to estimate the distribution of patients in modeled health states following CIK cell immunotherapy or no adjuvant therapy (9–11). Using the effectiveness data from the RWD, we tried to reflect current clinical practice for the early stage of HCC and to reduce the gap between the RCTs and the real-world setting Both RCT and RWD routine surveillance with CIK cell immunotherapy in an adjuvant setting for HCC were compared. At the median follow-up of 68.5 months for RCT and 28.0 months for RWD, investigators found statistically and clinically significant improvements in RFS (9–11). The patient characteristics and main results of both RCT and RWD were summarized in **Supplementary Table 1**.

RFS at a specific time was defined as the proportion of patients staying in the recurrence-free state (**Figure 1B**). The fraction of patients with recurrence was the difference between the OS and RFS (OS-RFS). The proportions of curable and incurable recurrence states were obtained from the RCTs to differentiate between patients receiving curative and noncurative treatment (**Table 1**). The incidence of curative treatment was estimated from the individual patient data reported in the RCTs. With regard to the distribution of patients who died, [1-OS] was adapted, as shown in **Figure 1B**.

**Table 1:** Model input parameters.

Model input	Value	PSA distribution	Sources
**Patient characteristics**			
Starting age, years	55		Lee et al. (9)
Proportion of curable recurrence, %			Lee et al.^a^ (10)
Treatment	56.6		
Control	47.8		
**Incidence rate of curative treatments (/person-year), mean (95% CI)**			Lee et al.^a^ (10)
Resection	0.0888 (0.0385, 0.1390)	Normal	
Radiofrequency ablation	0.3033 (0.2105, 0.3962)	Normal	
Percutaneous ethanol injection	0.2071 (0.1304, 0.2839)	Normal	
Liver transplantation	0.0148 (0.0000, 0.0353)	Normal	
**Utilities**			
Health state utility (95% CI)			Pollom et al. (16)
Recurrence-free state	0.88 (0.85, 0.92)	Beta	
Curable recurrence	0.88 (0.85, 0.92)	Beta	
Incurable recurrence	0.40 (0.32, 0.48)	Beta	
Treatment-related disutility			Ock et al.^b^ (17)
Resection	- 0.26		
Radiofrequency ablation	- 0.33		
Percutaneous ethanol injection	- 0.33		
Transplantation	- 0.21		
**Costs (USD), mean (SD)** **^c^**			
Treatment cost (per injection)	3,807		
Health state cost (per cycle)			
Recurrence-free state^d^	211 (21)	Gamma	Micro-costing
Curable recurrence^d^	211 (21)	Gamma	Micro-costing
Incurable recurrence	2,505 (711)	Gamma	HIRA data
Event cost for curative treatments			HIRA data
Resection	8,082 (3,015)	Gamma	
Radiofrequency ablation	2,085 (1,039)	Gamma	
Percutaneous ethanol injection	1,640 (1,282)	Gamma	
Liver transplantation	67,142 (23,888)	Gamma	
End-of-life cost^d^	6,798 (679)	Gamma	Yang, (18)

CI, confidence interval; HIRA, Health Insurance Review & Assessment Service of Korea; PSA, probabilistic sensitivity analysis; SD, standard deviation.

a Derived from post-hoc analysis of recurrence data from phase III trial (Lee et al., 2015 & Lee et al., 2018).

b Converted to disutility from Ock et al., 2017.

c 1 USD = 1,166.51 KRW.

d Standard deviation is assumed to be 10% of the mean value.

To extrapolate patient survival beyond the duration of the clinical trial, parametric survival curves were fitted to our model: exponential, Weibull, generalized gamma, log-normal, and log-logistic. They were modeled jointly for each treatment group, and the best fit was determined using the Akaike information criterion, Bayesian information criterion, plausibility of the estimated long-term survival, and expert opinion (**Supplementary Table 2** and **Supplementary Figures 1, 2**) (12, 19). The parameter estimates for survival curves are presented in **Supplementary Table 3**.

### Cost and Utility

We considered direct medical costs, which were represented in the model either as episodic costs for curative treatments and death (end-of-life) or state-based costs, in terms of the healthcare system perspective. The input costs, presented in USD in 2020 (1 USD = 1,166.51 KRW), are shown in **Table 1**. The cost data before 2020 were corrected for inflation using the national inflation calculator.

Treatment-specific costs in the recurrence-free state were estimated based on healthcare utilization from RCT and the unit cost from the reimbursement price list from HIRA. In accordance with the label of CIK cell immunotherapy, treatment cost was considered for a total of 16 times at the price proposed by the manufacturer.

The medical costs for recurrence were obtained from the HIRA National Patient Sample (HIRA-NPS-2016-0106) data. The cost analysis of HIRA-NPS data was approved by the institutional review board of Sungkyunkwan University. The HIRA-NPS data are representative of the South Korean population, which includes approximately 3% of the total population (20, 21). From the HIRA-NPS data, we extracted the medical costs per episode of each curative treatments (i.e., resection, RFA, PEI, and transplantation) and noncurative treatments (i.e., TACE, external-beam radiation therapy, and systemic therapy with sorafenib or cytotoxic chemotherapy) from patients with the main diagnosis code of HCC (C22.0 of ICD-10, International Classifications of Disease 10th version) in 2016. The detailed procedure codes which were used to estimate the medical costs for each treatment were presented in **Supplementary Table 4**. For the curable recurrence state, the cost for each curative treatment was individually applied according to the incidence of curative treatment in the model. For the incurable recurrence state, the state-based cost was calculated by multiplying the cost of noncurative treatment by the frequency obtained from the RCT. The end-of-life costs were adapted from a previous study that observed end-of-life costs for patients with cancer (18).

We performed a systematic literature review using PubMed and the Cochrane library to obtain utility values for each health state in our model (16, 17). Utility is a number between 0 (death) and 1 (perfect health), which is used to calculate QALY by taking length and quality of life into consideration (22). A detailed list of the utilities and disutilities included in the model are shown in **Table 1**.

### Analysis

The main output of this study was the incremental cost-effectiveness ratio (ICER), which was calculated by dividing the incremental costs by the incremental LYs and QALYs between the CIK cell immunotherapy group and no adjuvant therapy group. The cost-effectiveness was interpreted using ICER at the willingness-to-pay (WTP) threshold of $50,000/QALY.

Furthermore, one-way deterministic and probabilistic sensitivity analyses were performed to explore the robustness of the results by varying the parameter values and assumptions. For the one-way sensitivity analysis, clinical variables (parametric survival distribution to OS and RFS, and the proportion of patients receiving curative treatment), utility weights, medical costs, analytic perspective, time horizon, and discount rate were changed. Medical costs in other countries were also applied to the sensitivity analysis and are presented in **Supplementary Table 5** (23–25).

For the probabilistic sensitivity analysis, a comprehensive estimate of the uncertainty around the results was calculated using simultaneous random sampling of input parameters. The range and distribution of the parameters are listed in **Table 1** and **Supplementary Table 2**. The probabilistic sensitivity analysis consisted of 1,000 iterations with random values generated according to the range or distribution of each parameter included in the model. In addition, a cost-effectiveness acceptability curve (CEAC) was constructed to show the likelihood of the intervention being cost-effective according to the various WTP thresholds.

## Results

**Figure 2:**
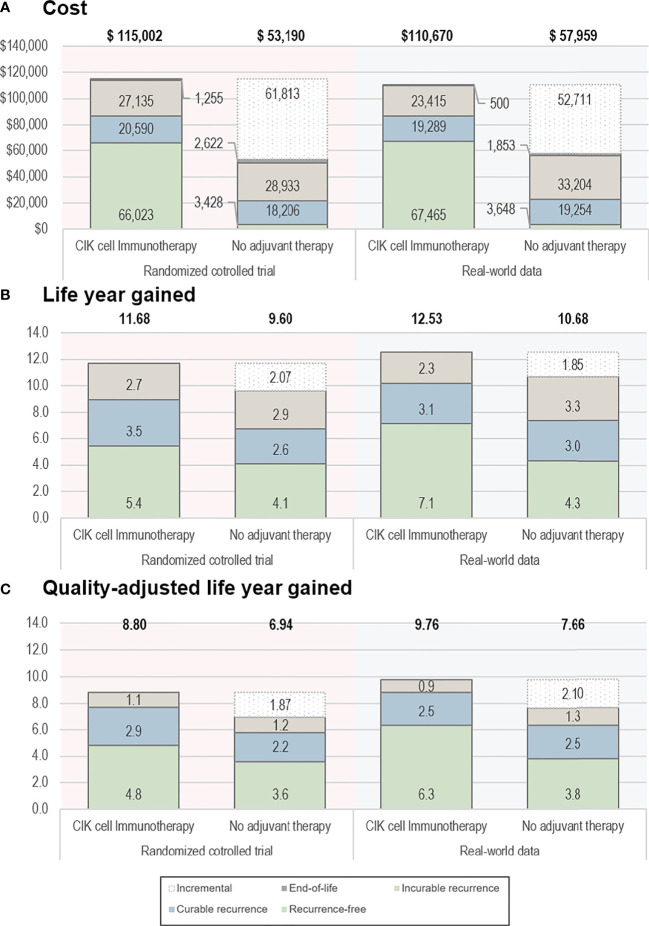
Base-case results. **(A)** Cost, **(B)** life-year gained, and **(C)** quality-adjusted life-year gained. The bold text indicates the total value estimated from the analyses. CIK, cytokine-induced killer.

Based on the RCT data, the CIK cell immunotherapy resulted in 11.68 LYs and 8.80 QALYs per patient costing $115,002, whereas no adjuvant therapy resulted in 9.60 LYs and 6.94 QALYs per patient costing $53,190 (**Table 2**). Throughout the time horizon, the incremental LYs was 2.07 years, and the patients treated with CIK cell immunotherapy remained in the recurrence-free state longer than the patients without treatment (5.43 vs. 4.07 years, **Figure 2**). For treatment costs, a substantial difference between the two interventions was observed in the recurrence-free state. The incremental QALYs was 1.87 costing $61,813, and the ICER was $33,077/QALY.

**Table 2:** Result of the cost-effectiveness analysis.

	CIK cell Immunotherapy	No adjuvant therapy	Incremental	ICER ($/LY or $/QALY)
**Based on RCT data**				
Cost	$115,002	$53,190	$61,813	
LY	11.68	9.60	2.07	$29,791
QALY	8.80	6.94	1.87	$33,077
**Based on RWD**				
Cost	$110,670	$57,959	$52,711	
LY	12.53	10.68	1.85	$28,437
QALY	9.76	7.66	2.10	$25,107

CIK, cytokine-induced killer cell; LY, life-year; QALY, quality-adjusted life-year; ICER, Incremental cost-effectiveness ratio; RCT, randomized controlled trial; RWD, real-world data.

Based on the RWD, the CIK cell immunotherapy resulted in 12.53 LYs and 9.76 QALYs per patient with a treatment cost of $110,670, whereas no adjuvant therapy resulted in 10.68 LYs and 7.66 QALYs per patient costing $57,959. The incremental life-years gained was 1.85, and the incremental QALY was 2.10, resulting in an ICER of $25,107/QALY. The ICERs estimated based on RCT data and RWD were both less than $50,000, which showed the cost-effectiveness of CIK cell immunotherapy.

**Figure 3:**
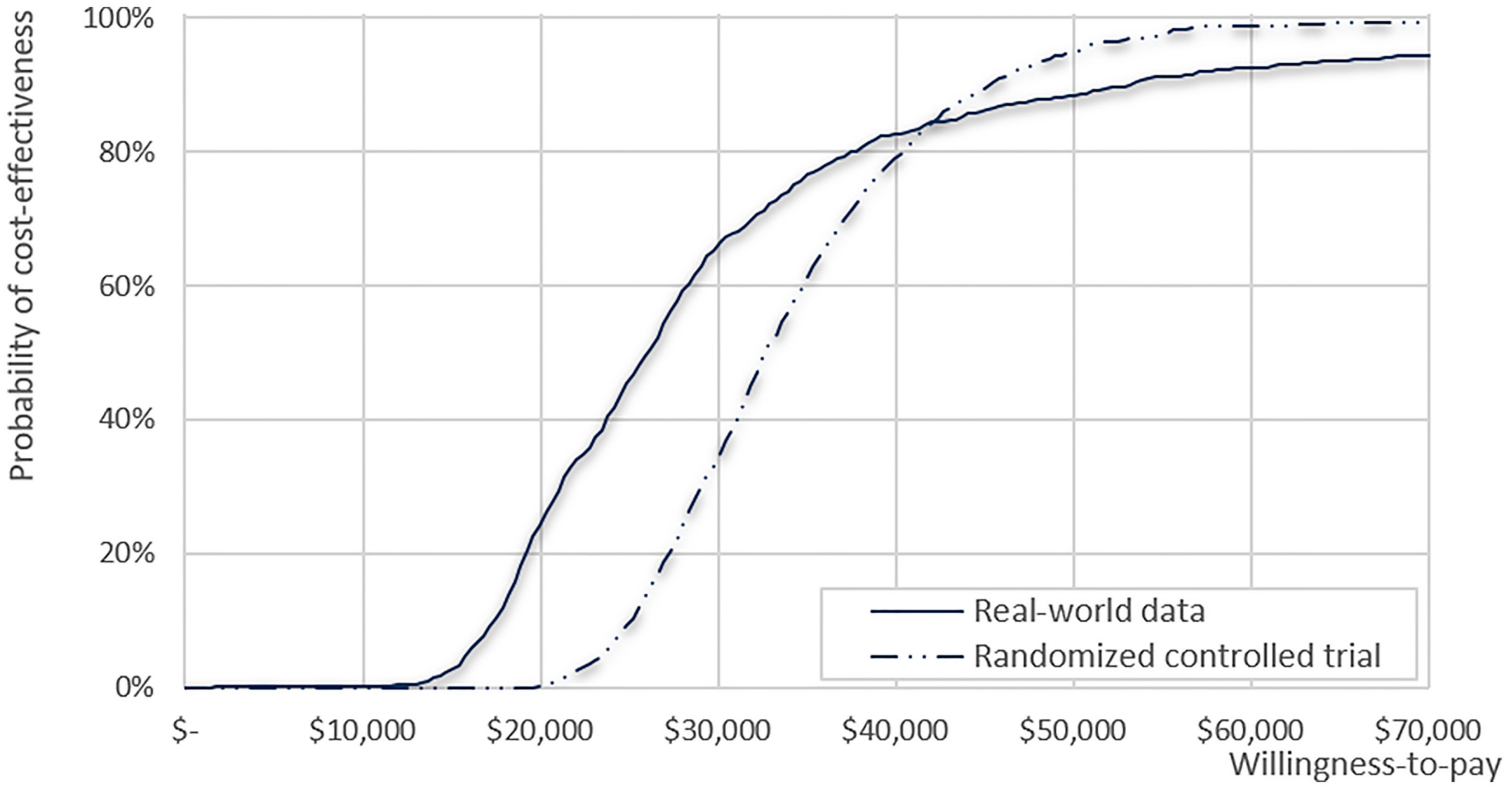
Cost-effectiveness acceptability curve for clinical data from the real-world data and randomized clinical trial.

The results of the deterministic sensitivity analysis are presented in **Supplementary Figure 3** and **Table 3**, showing the 10 most sensitive input parameters, given RCT data and RWD. The parameter that most influenced the ICER was the time horizon. Regarding other parameters, including productivity loss in the analysis (societal perspective) resulted in lower ICER ($ 25,562), whereas applying medical costs from Italy and the USA showed the robustness of the study results with ICER ranging from $34,141–$38,425 (**Supplementary Table 3**). All ICERs based on the RWD were lower than those based on the RCTs. The CEAC calculated from our model showed that the likelihood of CIK cell immunotherapy being cost-effective was 95% and 88% based on RCT and RWD, respectively, with a WTP threshold of $50,000 (**Figure 3**). For WTP values below $42,350, the cost-effectiveness acceptability based on RWD was higher than that based on RCT, but the trend reversed for WTP values beyond $42,350.

**Table 3:** Deterministic sensitivity analyses..

Scenario	ICER (US$/QALY)	
	RCT	RWD
**Base-case**	33,077	25,107
**Clinical outcomes**		
Survival^*^		
RFS [Best case; Weibull (RCT), Weibull (RWD)]	31,260	22,948
RFS [Worst case; Log-normal (RCT), Generalized gamma (RWD)]	33,077	31,695
OS [Best case; Weibull (RCT), Generalized gamma (RWD))	31,009	22,587
OS [Worst case; Generalized gamma (RCT), Log-normal (RWD)]	38,831	27,545
** Proportion of curable recurrence**		
Considering a portion of TACE as a curative treatment (CIK 75.8% vs No Tx 72.6%)	36,293	29,237
** Health-related quality of life**		
** Health state utilities**		
Cancer free and incurable recurrence state (Upper bound)	31,876	24,978
Cancer free and incurable recurrence state (Lower bound)	33,971	25,236
**Costs**		
** Medical costs from other healthcare systems**		
The USA [derived from Cardier et al., (23)]	38,425	9,505
France [derived from Cardier et al., (23)]	34,617	25,626
Italy [derived from Rognoni et al., (24)]	34,141	22,197
** End-of-life cost**		
Upper bound (+20%)	32,930	24,695
Lower bound (–20%)	33,223	25,187
**Analytic perspective (Societal perspective)**		
Including productivity loss cost	25,562	19,858
**Model parameters**		
Time horizon (15 years)	41,628	32,730
Time horizon (25 years)	28,799	21,263
Annual discount rate (3%)	27,617	20,336
Annual discount rate (7.5%)	40,926	31,973

ICER, incremental cost-effectiveness ratio; RCT, randomized controlled trial; RWD, real-world data; RFS, recurrence-free survival; OS, overall survival; TACE, transarterial chemoembolization.

^*^To see the uncertainty from the selected survival curve, we carried out a sensitivity analysis that calculated the lowest (best case) and the highest (worst case) ICER by applying each parametric survival distribution to OS and RFS.

## Discussion

This study demonstrated that adjuvant CIK cell immunotherapy in patients who received curative treatment for HCC is cost-effective as compared with no adjuvant therapy. By applying data from RCTs and an extended follow-up study, we showed that the higher LYs and QALYs gained from receiving CIK cell immunotherapy compared with no adjuvant therapy resulted from prolonged survival, reduced recurrence of HCC, and better prognosis of recurrence (9, 10). Furthermore, decreased medical expenses for the treatment of incurable recurrent HCC partially offset the considerable treatment cost of CIK cell immunotherapy in patient with curable recurrent HCC. Consequently, adjuvant CIK cell immunotherapy could be a cost-effective option based on a WTP threshold of US$ 50,000/QALY.

The simulation results using RWD, where CIK cell immunotherapy prolonged the RFS of patients with HCC, showed further improvement in cost-effectiveness, which was attributed to more favorable survival for CIK cell immunotherapy than that from the RCT data. The cost-effectiveness model using RWD shows that more patients stayed in recurrence-free health state due to better RFS than that from RCT. Therefore, scenario using RWD incurred less medical expenditure and have more health-related quality of life due to prolonged recurrence-free survival, which means the cost-effectiveness results using RWD were better than that from RCT data.

In the RCT, adjuvant CIK cell immunotherapy was more effective at reducing the rate of early recurrence within the first 24 months (which is mainly associated with residual tumor cells) than late recurrence beyond 24 months (which is mainly related to *de novo* carcinogenesis from diseased liver). Based on this result, the authors of the RWD study suggested that a shorter follow-up duration of RWD (28.0 months) than that of RCT (68.5 months) may be associated with lower HR of tumor recurrence (0.42 vs 0.67) (9–11). However, it should be noticed that the baseline characteristics of patients were worse with higher tumor stage and larger tumor size in RWD than in RCT. These baseline characteristics might be unfavorable to RWD as CIK cell treatment is expected to be more effective in patients with less tumor burden (9). Thus, the lower HR of tumor recurrence or death in RWD is not simply explainable with currently available data and further real-world studies are warranted.

Our findings provide evidence for generalizing the effectiveness of RCTs conducted in controlled populations in the real-world population. The participants in the RCT, who were included following clear inclusion and exclusion criteria and treated following a well-defined schedule, may not represent patients in real-world practice (26). Thus, the validation of the effectiveness of CIK cell adjuvant therapy in RWD is essential, and the benefit of RFS is reproducible. Furthermore, our findings are meaningful in that a more beneficial economic value of CIK cell immunotherapy could be obtained in real-world clinical practice. In addition, the cost-effectiveness results from both RCT and RWD can support decision-making on introducing CIK cell immunotherapy in an adjuvant setting.

The analysis conducted under the societal perspective considering productivity loss was remarkable compared to that conducted from the healthcare system perspective. HCC occurs more frequently in men and is most frequently diagnosed among people aged 55–64 years who are economically active, and also occurs at a younger age, especially in Asia and sub-Saharan Africa, where HBV is endemic, than in other regions where HBV is not a predominant etiology of HCC (27–30). As the socioeconomic burden of disease because of premature death is substantial, preventing recurrence in such patients would reduce productivity loss in society. In South Korea, an HBV-endemic country, HCC was the second-highest cause of cancer-related death (21.5 per 100.000 population) in 2016 following lung cancer but resulted in the highest economic burden (USD 3.144 million) in 2010 among all cancers (31). It is a disappointing outcome considering that the nationwide regular surveillance program is now working well and approximately 40% of new HCC cases are diagnosed at a very early or early stage, where potentially curative treatment can be applied (32). Accordingly, the urgent need for cost-effective adjuvant therapy should be highlighted again.

Our findings on the cost-effectiveness of CIK cell immunotherapy, which prevents the recurrence of HCC, align with the results of previous cost-effectiveness studies on adjuvant therapies. Although there is no guideline-suggested adjuvant therapy for patients with HCC, several RCTs were conducted for adjuvant therapy with an effective drug used in the management of advanced HCC, such as sorafenib (33, 34). Because our study was the first to demonstrate the cost-effectiveness of treatment to prevent recurrence in HCC, there was no previous study to report the cost-effectiveness of adjuvant therapy in patients with HCC who underwent a potentially curative treatment. However, there have been several studies on other types of cancers, in which cost-effectiveness was verified by preventing recurrence or relapse (35–37). Our results emphasize the importance of preventing recurrence after successful primary therapy, which is in line with previously reported findings.

Our research has significant strengths, although decision-analytic models have limitations regarding the input parameters applied in the model. First, our original model was designed to reflect the prognosis of HCC recurrence, which is not generally considered in the conventional three-state model. To reflect the heterogeneous prognosis of recurrence, we conducted a *post-hoc* analysis of subsequent treatments (curative and noncurative treatments) from the phase III clinical trial, followed by the proportion of curable recurrence that we applied to our original model. Therefore, we have improved the model plausibility by reflecting the different prognoses of recurrence and health-related quality of life of patients. In addition, we demonstrated the robustness of cost-effectiveness by performing probabilistic and deterministic sensitivity analyses. Most of the input parameters were derived from the patient-level data of the phase III trial, and the remaining input parameters were verified by clinical experts. Moreover, we applied a wide range of costs and effectiveness to our model to assess inherent uncertainties.

Although our study considered various ranges of uncertainty, there were some limitations. First, the applied costs were obtained from the Korean healthcare system. Therefore, the costs were potentially lower than those in other developed countries, such as the USA. However, cost-effectiveness evaluations focus on the ‘incremental’ costs and effectiveness of CIK cell immunotherapy, and because the unit medical costs affected both the intervention and comparator in our model, the impact of lower costs in South Korea on the results would be limited. Even if the local cost data were replaced with those in other developed countries, including the USA, France, and Italy, the estimated ICERs from those scenarios ($34,000–$38,000/QALY) were similar to our base-case analysis results and were similar to or lower than each country’s gross domestic product (GDP) per capita. Because the cost-effectiveness threshold for anticancer drugs is generally accepted as approximately twice the GDP per capita, CIK cell immunotherapy would be a cost-effective alternative in all of these countries as well. In addition, the discount rate applied to our model was 5%, which was slightly higher than that in other developed countries; thus, the cost-effectiveness of CIK cell immunotherapy was analyzed conservatively because the long-term effectiveness and offset treatment costs of incurable recurrent HCC were underestimated in the base-case setting.

Second, survival data from RWD study has a relatively shorter observation period than RCTs, and there were some differences in the baseline characteristics among RWD and RCTs. This may increase the uncertainty of modeled survival output using RWD. Therefore, we simulated various parametric survival functions in sensitivity analyses to alleviate the uncertainty caused by short observation periods and different patient characteristics from RCTs, and presented the minimum and maximum values of ICER according to various survival functions in the tornado diagram (**Supplementary Figure 3**). As a result, our model showed robust results even when using RWD data. Finally, the proportion of curable recurrent HCC was calculated based on operational definitions using the subsequent treatment data of patients obtained from RCTs. For example, each recurrence treated with TACE was classified as incurable recurrence. However, there could be a curative TACE for patients who are not eligible for other curative treatments, which could lead to an underestimation of the proportion of curable recurrence. Even if we adopted the most generous criteria for classifying the curability of recurrence, the ICER increased by only 7% from the base-case analysis, and CIK cell immunotherapy was still cost-effective.

In conclusion, we showed that receiving CIK immunotherapy was more cost-effective than no adjuvant therapy in patients with HCC who underwent a potentially curative treatment, attributed to the prolonged survival, reduced recurrence of HCC, and better prognosis of recurrence, and it could be even more cost-effective in the real-world clinical practice.

## Data Availability Statement

The raw data supporting the conclusions of this article will be made available by the authors, without undue reservation.

## Author Contributions

H-LK, E-KL, and J-HL contributed to conception and design of the study. J-HL organized the database. H-LK, S-HK, and J-YC performed the statistical analyses and interpreted results. J-YC and S-HK contributed equally to this work and deserve co-first authorship. H-LK and J-HL are co-corresponding authors. All authors contributed to the article and approved the submitted version.

## Funding

This study was supported by GC Cell Corp (Yongin, Korea). The sponsor was not involved in the study design, collection, analysis, interpretation of data, the writing of this article or the decision to submit it for publication.

## Conflict of Interest

E-KL report receiving grants from Green Cross Cell Corp during the conduct of the study. J-HL reports a research grant and lecture fee from Green Cross Cell Corp. H-LK has received research grant and lecture fee from Green Cross Cell Corp.

The remaining authors declare that the research was conducted in the absence of any commercial or financial relationships that could be construed as a potential conflict of interest.

## Publisher’s Note

All claims expressed in this article are solely those of the authors and do not necessarily represent those of their affiliated organizations, or those of the publisher, the editors and the reviewers. Any product that may be evaluated in this article, or claim that may be made by its manufacturer, is not guaranteed or endorsed by the publisher.

## References

[1] Bray F Ferlay J Soerjomataram I Siegel RL Torre LA Jemal A. Global Cancer Statistics 2018: GLOBOCAN Estimates of Incidence and Mortality Worldwide for 36 Cancers in 185 Countries. CA Cancer J Clin (2018) 68(6):394–424. doi: 10.3322/caac.21492 30207593

[2] Park JW Chen M Colombo M Roberts LR Schwartz M Chen PJ. Global Patterns of Hepatocellular Carcinoma Management From Diagnosis to Death: The BRIDGE Study. Liver Int (2015) 35(9):2155–66. doi: 10.1111/liv.12818 PMC4691343 25752327

[3] European Association for the Study of the Liver EASL Clinical Practice Guidelines: Management of Hepatocellular Carcinoma. J Hepatol (2018) 69(1):182–236. doi: 10.1016/j.jhep.2018.03.019 29628281

[4] Llovet JM Di Bisceglie AM Bruix J Kramer BS Lencioni R Zhu AX. Design and Endpoints of Clinical Trials in Hepatocellular Carcinoma. J Natl Cancer Inst (2008) 100(10):698–711. doi: 10.1093/jnci/djn134 18477802

[5] Shah SA Cleary SP Wei AC Yang I Taylor BR Hemming AW. Recurrence After Liver Resection for Hepatocellular Carcinoma: Risk Factors, Treatment, and Outcomes. Surgery (2007) 141(3):330–9. doi: 10.1016/j.surg.2006.06.028 17349844

[6] Sempokuya T Wong LL. Ten-Year Survival and Recurrence of Hepatocellular Cancer. Hepatoma Res (2019) 5:38. doi: 10.20517/2394-5079.2019.013 31701016 PMC6836870

[7] Schmidt-Wolf IG Negrin RS Kiem HP Blume KG Weissman IL. Use of a SCID Mouse/Human Lymphoma Model to Evaluate Cytokine-Induced Killer Cells With Potent Antitumor Cell Activity. J Exp Med (1991) 174(1):139–49. doi: 10.1084/jem.174.1.139 PMC2118875 1711560

[8] Zhang Y Schmidt-Wolf IGH. Ten-Year Update of the International Registry on Cytokine-Induced Killer Cells in Cancer Immunotherapy. J Cell Physiol (2020) 235(12):9291–303. doi: 10.1002/jcp.29827 32484595

[9] Lee JH Lee JH Lim YS Yeon JE Song TJ Yu SJ. Adjuvant Immunotherapy With Autologous Cytokine-Induced Killer Cells for Hepatocellular Carcinoma. Gastroenterology (2015) 148(7):1383–91.e6. doi: 10.1053/j.gastro.2015.02.055 25747273

[10] Lee JH Lee JH Lim YS Yeon JE Song TJ Yu SJ. Sustained Efficacy of Adjuvant Immunotherapy With Cytokine-Induced Killer Cells for Hepatocellular Carcinoma: An Extended 5-Year Follow-Up. Cancer Immunol Immunother (2019) 68(1):23–32. doi: 10.1007/s00262-018-2247-4 30232520 PMC6326973

[11] Yoon JS Song BG Lee JH Lee HY Kim SW Chang Y. Adjuvant Cytokine-Induced Killer Cell Immunotherapy for Hepatocellular Carcinoma: A Propensity Score-Matched Analysis of Real-World Data. BMC Cancer (2019) 19(1):523. doi: 10.1186/s12885-019-5740-z 31151419 PMC6543598

[12] Woods BS Sideris E Palmer S Latimer N Soares M. Partitioned Survival and State Transition Models for Healthcare Decision Making in Oncology: Where Are We Now? Value Health (2020) 23(12):1613–21. doi: 10.1016/j.jval.2020.08.2094 33248517

[13] Marrero JA Kulik LM Sirlin CB Zhu AX Finn RS Abecassis MM. Diagnosis, Staging, and Management of Hepatocellular Carcinoma: 2018 Practice Guidance by the American Association for the Study of Liver Diseases. Clin Liver Dis (Hoboken) (2019) 13(1):1. doi: 10.1002/cld.802 29624699

[14] The Health Insurance Review and Assessment Service (HIRA) Guidelines for Economic Evaluation of Pharmaceuticals in Korea. Wonju: Health Insurance Review & Assessment Service (2011).

[15] Husereau D Drummond M Petrou S Carswell C Moher D Greenberg D. Consolidated Health Economic Evaluation Reporting Standards (CHEERS)–Explanation and Elaboration: A Report of the ISPOR Health Economic Evaluation Publication Guidelines Good Reporting Practices Task Force. Value Health (2013) 16(2):231–50. doi: 10.1016/j.jval.2013.02.002 23538175

[16] Pollom EL Lee K Durkee BY Grade M Mokhtari DA Wahl DR. Cost-Effectiveness of Stereotactic Body Radiation Therapy Versus Radiofrequency Ablation for Hepatocellular Carcinoma: A Markov Modeling Study. Radiology (2017) 283(2):460–8. doi: 10.1148/radiol.2016161509 PMC5410949 28045603

[17] Ock M Lim SY Lee HJ Kim SH Jo MW. Estimation of Utility Weights for Major Liver Diseases According to Disease Severity in Korea. BMC Gastroenterol (2017) 17(1):103. doi: 10.1186/s12876-017-0660-3 28870162 PMC5584479

[18] Yang SY Park SK Kang HR Kim HL Lee EK Kwon SH. Haematological Cancer Versus Solid Tumour End-of-Life Care: A Longitudinal Data Analysis. BMJ Support Palliat Care (2020). doi: 10.1136/bmjspcare-2020-002453 33376113

[19] Hoyle MW Henley W. Improved Curve Fits to Summary Survival Data: Application to Economic Evaluation of Health Technologies. BMC Med Res Methodol (2011) 11:139. doi: 10.1186/1471-2288-11-139 21985358 PMC3198983

[20] Peabody JW Lee SW Bickel SR. Health for All in the Republic of Korea: One Country’s Experience With Implementing Universal Health Care. Health Policy (1995) 31(1):29–42. doi: 10.1016/0168-8510(94)00669-6 10140361

[21] Kim L Kim JA Kim S. A Guide for the Utilization of Health Insurance Review and Assessment Service National Patient Samples. Epidemiol Health (2014) 36:e2014008. doi: 10.4178/epih/e2014008 25078381 PMC4151963

[22] Weinstein MC Torrance G McGuire A. QALYs: The Basics. Value Health (2009) 12(Suppl 1):S5–9. doi: 10.1111/j.1524-4733.2009.00515.x 19250132

[23] Cadier B Bulsei J Nahon P Seror O Laurent A Rosa I. Early Detection and Curative Treatment of Hepatocellular Carcinoma: A Cost-Effectiveness Analysis in France and in the United States. Hepatol (Baltimore Md) (2017) 65(4):1237–48. doi: 10.1002/hep.28961 28176349

[24] Rognoni C Ciani O Sommariva S Tarricone R. Real-World Data for the Evaluation of Transarterial Radioembolization Versus Sorafenib in Hepatocellular Carcinoma: A Cost-Effectiveness Analysis. Value Health (2017) 20(3):336–44. doi: 10.1016/j.jval.2016.09.2397 28292478

[25] Tangka FK Subramanian S Sabatino SA Howard DH Haber S Hoover S. End-Of-Life Medical Costs of Medicaid Cancer Patients. Health Serv Res (2015) 50(3):690–709. doi: 10.1111/1475-6773.12259 25424134 PMC4450925

[26] May GS DeMets DL Friedman LM Furberg C Passamani E. The Randomized Clinical Trial: Bias in Analysis. Circulation (1981) 64(4):669–73. doi: 10.1161/01.CIR.64.4.669 7023743

[27] White DL Thrift AP Kanwal F Davila J El-Serag HB. Incidence of Hepatocellular Carcinoma in All 50 United States, From 2000 Through 2012. Gastroenterology (2017) 152(4):812–20.e5. doi: 10.1053/j.gastro.2016.11.020 27889576 PMC5346030

[28] Kennedy K Graham SM Arora N Shuhart MC Kim HN. Hepatocellular Carcinoma Among US and Non-US-Born Patients With Chronic Hepatitis B: Risk Factors and Age at Diagnosis. PloS One (2018) 13(9):e0204031. doi: 10.1371/journal.pone.0204031 30252863 PMC6155504

[29] National Cancer Institute Surveillance, Epidemiology, and End Results Program. Cancer Stat Facts: Liver and Intrahepatic Bile Duct Cancer. (2020). Available at: https://seer.cancer.gov/statfacts/html/livibd.html [November 24. 2021].

[30] Zhu RX Seto WK Lai CL Yuen MF. Epidemiology of Hepatocellular Carcinoma in the Asia-Pacific Region. Gut Liver (2016) 10(3):332–9. doi: 10.5009/gnl15257 PMC4849684 27114433

[31] Korean Liver Cancer Association; National Cancer Center. 2018 Korean Liver Cancer Association-National Cancer Center Korea Practice Guidelines for the Management of Hepatocellular Carcinoma. Gut Liver (2019) 13(3):227–99. doi: 10.5009/gnl19024 PMC6529163 31060120

[32] Chon YE Lee HA Yoon JS Park JY Kim BH Lee IJ. Hepatocellular Carcinoma in Korea Between 2012 and 2014: An Analysis of Data From the Korean Nationwide Cancer Registry. J Liver Cancer (2020) 20(2):135–47. doi: 10.17998/jlc.20.2.135 PMC10035678 37384317

[33] Bruix J Takayama T Mazzaferro V Chau GY Yang J Kudo M. Adjuvant Sorafenib for Hepatocellular Carcinoma After Resection or Ablation (STORM): A Phase 3, Randomised, Double-Blind, Placebo-Controlled Trial. Lancet Oncol (2015) 16(13):1344–54. doi: 10.1016/S1470-2045(15)00198-9 26361969

[34] Zhu XD Li KS Sun HC. Adjuvant Therapies After Curative Treatments for Hepatocellular Carcinoma: Current Status and Prospects. Genes Dis (2020) 7(3):359–69. doi: 10.1016/j.gendis.2020.02.002 PMC7452398 32884990

[35] Seferina SC Ramaekers BLT de Boer M Dercksen MW van den Berkmortel F van Kampen RJW. Cost and Cost-Effectiveness of Adjuvant Trastuzumab in the Real World Setting: A Study of the Southeast Netherlands Breast Cancer Consortium. Oncotarget (2017) 8(45):79223–33. doi: 10.18632/oncotarget.16985 PMC5668034 29108301

[36] Millar JA Millward MJ. Cost Effectiveness of Trastuzumab in the Adjuvant Treatment of Early Breast Cancer: A Lifetime Model. Pharmacoeconomics (2007) 25(5):429–42. doi: 10.2165/00019053-200725050-00006 17488140

[37] Hisashige A Sasako M Nakajima T. Cost-Effectiveness of Adjuvant Chemotherapy for Curatively Resected Gastric Cancer With S-1. BMC Cancer (2013) 13:443. doi: 10.1186/1471-2407-13-443 24079752 PMC3816158

