# Supplementary material for: Cost-Effectiveness of Adjuvant Immunotherapy With Cytokine-Induced Killer Cell for Hepatocellular Carcinoma Based on a Randomized Controlled Trial and Real-World Data
Source: Front Oncol. 2021 Dec 3;11:728740. doi: 10.3389/fonc.2021.728740 (PMC8682810; doi:10.3389/fonc.2021.728740)
Supplement: Supplementary file 1 [file DataSheet_1.pdf]

frontiers

Cost-effectiveness of adjuvant immunotherapy with cytokine-induced killer cells for hepatocellular carcinoma based on a randomized controlled trial and real-world data

Jeong-Yeon Cho, Sun-Hong Kwon, Eui-Kyung Lee, Jeong-Hoon Lee, and Hye-Lin Kim

Table of contents

Supplementary Table 1 ……...……………….…………………………………………………..2
Supplementary Table 2 ……...……………….…………………………………………………..3
Supplementary Table 3 ……...……………….…………………………………………………..4
Supplementary Table 4 ……...……………….…………………………………………………..5
Supplementary Table 5 ……...……………….…………………………………………………..6
Supplementary Figure 1……..….……………….……………………………………………….7
Supplementary Figure 2……..….……………….……………………………………………….8
Supplementary Figure 3……..….……………….……………………………………………….9

Supplementary Table 1. Study comparison between RCT and RWD

	Randomized controlled trial*		Real-world data‡	
	CIK cell immunotherapy (n=114)	No adjuvant therapy (n=112)	CIK cell immunotherapy (n=59)	No adjuvant therapy (n=59)
**Patient characteristic**				
Male sex, N (%)	95 (83.3%)	91 (81.3%)	45 (76.3%)	46 (78.0%)
Age, years†	55.4 ± 8.2	56.4 ± 10.6	57.0 (48.5–62.0)	59.0 (52.0–65.0)
HCC stage, N (%)				
Stage I	98 (86.0%)	94 (83.9%)	25 (42.4%)	30 (50.8%)
Stage II	16 (14.0%)	18 (16.1%)	34 (57.6%)	29 (49.2%)
Number of HCC, N (%)				
< 3	112 (98.2%)	110 (98.2%)	57 (96.6%)	57 (96.6%)
≥ 3	2 (1.8%)	2 (1.8%)	2 (3.4%)	2 (3.4%)
Size of HCC, cm	1.8 (1.4–2.3)	2.3 (1.5–3.1)	2.9 (2.1–3.9)	2.3 (1.9–3.6)
Cirrhosis, N (%)	76 (66.7%)	70 (62.5%)	35 (59.3%)	31 (52.5%)
**Summary of result**				
Follow-up (months)	68.5 (45.0–82.2)		28.0 (22.9–42.3)	
Median RFS (months)	44.0	30.0	Not reached	29.8
Hazard ratio *(ref: No adjuvant therapy)*	0.67 (95% CI 0.48–0.94)		0.42 (95% CI, 0.22–0.80)	

* Ref. Lee et al., 2015 (1) & Lee et al., 2019 (2)
‡ Ref. Yoon et al., 2019 (3)
†Age was expressed as mean ± standard error in RCT, median with interquartile range in RWD, respectively.
PS, propensity score; CIK, Cytokine-induced killer; HCC, hepatocellular carcinoma; RFS, recurrence-free survival

Supplementary Table 2. Goodness-of-fit statistics for survival curves

Parametric survival curves	Recurrence-free survival		Overall survival	
	AIC	BIC	AIC	BIC
**Survival curves derived from randomized controlled trial (RCT)**				
Exponential	643.14	649.9	198.8	205.57
Weibull	641.01	651.15	196.51	206.66
Log-logistic	637.22	647.36	196.01	206.16
Log-normal	634.83	644.97	193.64	203.79
Generalized gamma	636.73	650.25	191.68	205.22
**Survival curves derived from real-world data (RWD)**				
Exponential	239.44	244.98	51.17	56.71
Weibull	241.44	249.75	53.06	61.37
Log-logistic	239.3	247.62	53.03	61.34
Log-normal	235.97	244.28	52.73	61.04
Generalized gamma	235.32	246.4	55.09	66.17

AIC, Akaike information criterion; BIC, Bayesian information criterion

Supplementary Table 3. Results of parametric estimation for survival curves

Model input	Value	PSA distribution	Sources
**Parameter for survival curves derived from randomized controlled trial (RCT)**			Lee et al., 2018[a]
Recurrence-free survival			
Best-fitted parametric distribution	Log-normal		
Log mean	3.3693	Normal	
Log SD	1.5794		
Coefficient for treatment *(ref. no adjuvant therapy)*	0.5349		
Overall survival			
Best-fitted parametric distribution	Log-normal		
Log mean	5.2917	Normal	
Log SD	1.2811		
Coefficient for treatment *(ref. no adjuvant therapy)*	0.8864		
**Parameter for survival curves derived from real-world data (RWD)**			Yoon et al., 2019[a]
Recurrence-free survival			
Best-fitted parametric distribution	Log-normal		
Log mean	3.5222	Normal	
Log SD	1.4788		
Coefficient for treatment *(ref. no adjuvant therapy)*	0.9933		
Overall survival			
Best-fitted parametric distribution	Exponential		
Shape	6.1329	Normal	
Coefficient for treatment *(ref. no adjuvant therapy)*	1.4715		

[a] Parameters were analyzed with patient-level data from Lee et al., (2018) or Yoon et al., (2019).(2, 3)

Supplementary Table 4. List of codes for cost analysis

Item	Code
**Main diagnosis code**	
Hepatocellular carcinoma	C22.0
**Procedure code**	
**Curative treatment**	
Liver resection	Q7221, Q7222, Q7223, Q7224
RFA	QZ841
PEI	M1773
Transplantation	Q8040, Q8041, Q8042, Q8043, Q8044, Q8045, Q8046, Q8047, Q8048, Q8049, Q8050
**Noncurative treatment**	
TACE	M6644
RT*	AZ101, AZ201, AZ301
Cytotoxic chemotherapy*	AZ100, AZ200, AZ300
Sorafenib	488001ATB

*The code for education fee was utilized to identify records of radiotherapy and cytotoxic chemotherapy.
RFA, radiofrequency ablation; PEI, percutaneous ethanol injection; TACE, transarterial chemoembolization; RT, radiotherapy

Supplementary Table 5. Costs in South Korea, USA, France, and Italy for one-way sensitivity analyses

Cost items	South Korea	USA[†]	France[†]	Italy[‡]
**Cost in cancer free state (per cycle)**				
Follow-up for HCC recurrence[§]	211	2,066	580	212
**Cost related to treatment (per event)**				
Liver resection	8,082	25,086	14,914	8,451
Radiofrequency ablation (RFA)	2,085	18,386	4,149	8,451
Percutaneous ethanol injection (PEI)	1,640	18,386[*]	4,149[*]	8,451
Liver transplantation	67,142	137,701	57,426	76,153
Transarterial chemoembolization (TACE)	3,165	25,961	4,219	4,536
Cytotoxic chemotherapy	2,465	3,444	4,185	4,499[*]
Radiation therapy	3,653	25,891[*]	4,208[*]	4,524
Sorafenib *(per cycle)*	6,163	62,610	10,114	11,876
**End-of-life cost**	6,798	26,062[⁑]	15,494[*]	8,780[*]
**Incremental cost-effectiveness ratio (per QALY)**	$ 33,077	$38,425	$ 34,617	$ 34,141

[†] Costs derived from Cardier et al., 2017.(4)
[‡] Costs derived from Rognoni et al., 2017.(5)
[§] Follow-up cost included oncologist consultation, laboratory tests and imaging costs
[*] Cost of items was estimated using the ratio of the country in which the item was presented; i.e., cost of percutaneous ethanol injection was estimated as same as cost of radiofrequency ablation since both cost are same in Italy.
[⁑] Costs derived from Tangka et al., 2015.(6)

Supplementary Figure 1. Parametric survival curves from the randomized controlled trial by distribution. (A) RFS of CIK cell immunotherapy group, (B) RFS of no adjuvant therapy group, (C) OS of CIK cell immunotherapy group and (D) OS of no adjuvant therapy group; RFS, Recurrence-free survival; OS, Overall survival; CIK, Cytokine-induced killer

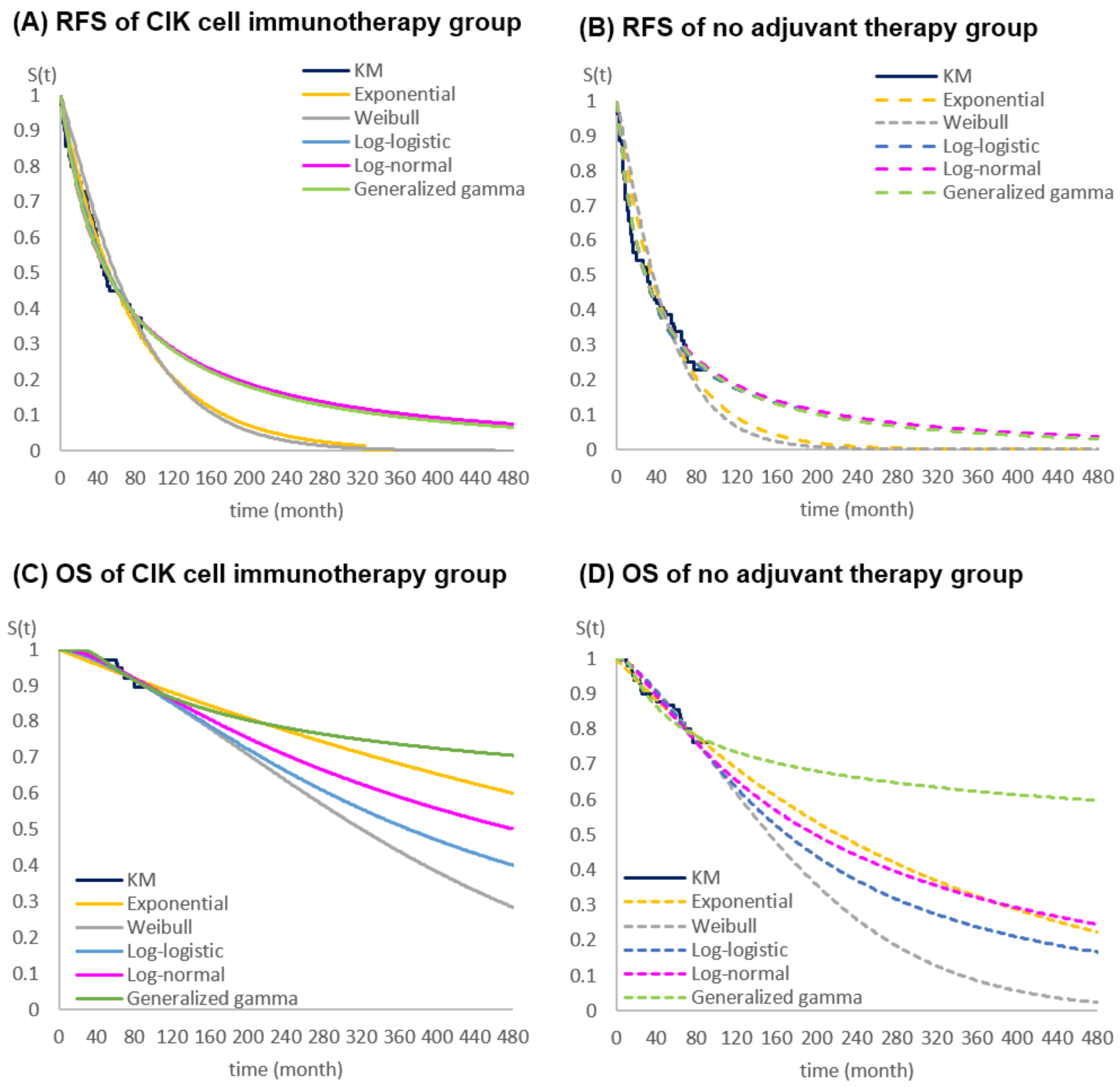

Supplementary Figure 2. Parametric survival curves from the real-world data by distribution. (A) RFS of CIK cell immunotherapy group, (B) RFS of no adjuvant therapy group, (C) OS of CIK cell immunotherapy group and (D) OS of no adjuvant therapy group; RFS, Recurrence-free survival; OS, Overall survival; CIK, Cytokine-induced killer

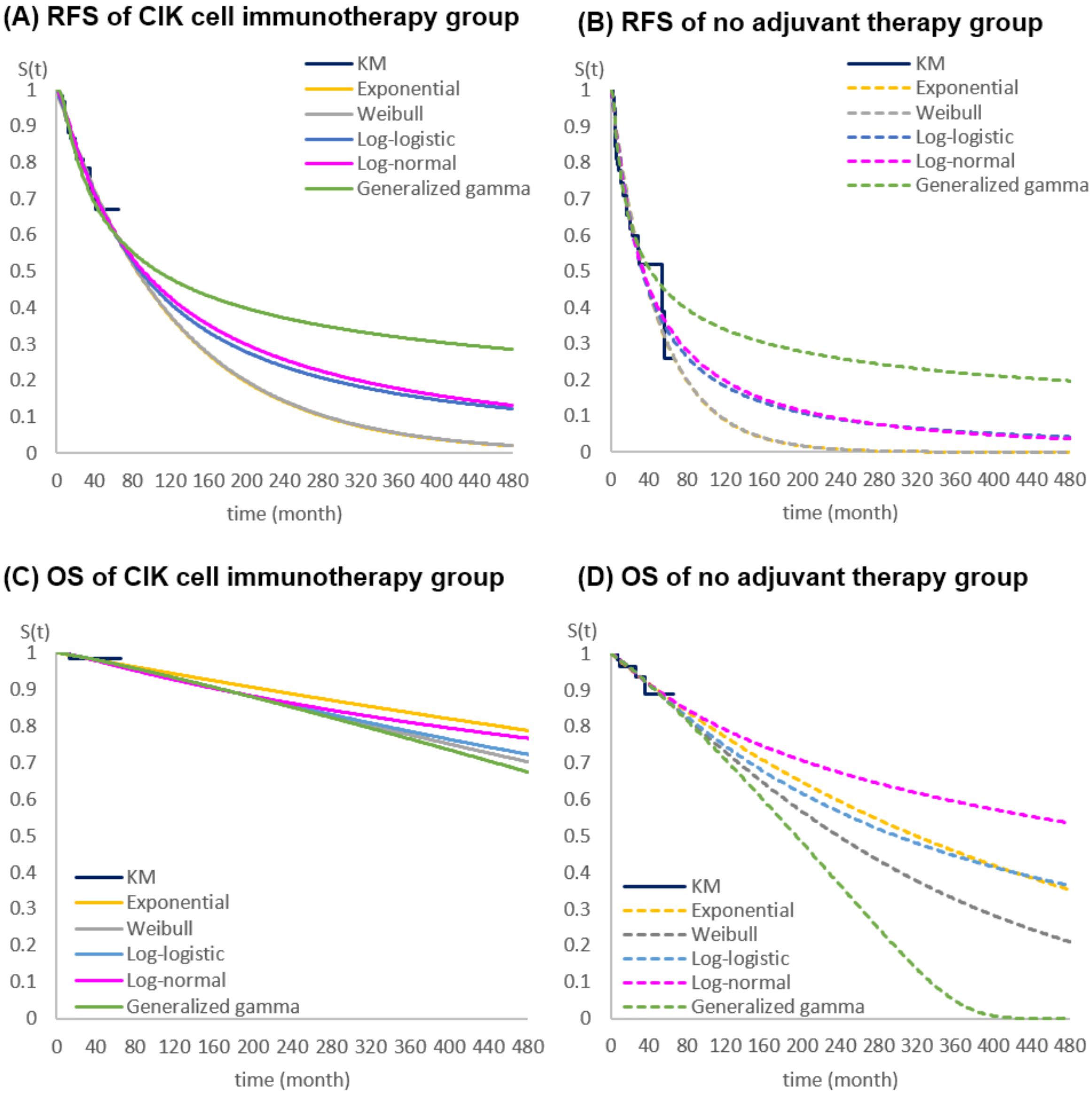

Supplementary Figure 3. One-way sensitivity analyses by clinical outcome source. (A) From the randomized controlled trial, and (B) From the real-world data ICER, Incremental cost-effectiveness ratio; OS, Overall survival; RFS, Recurrence-free survival

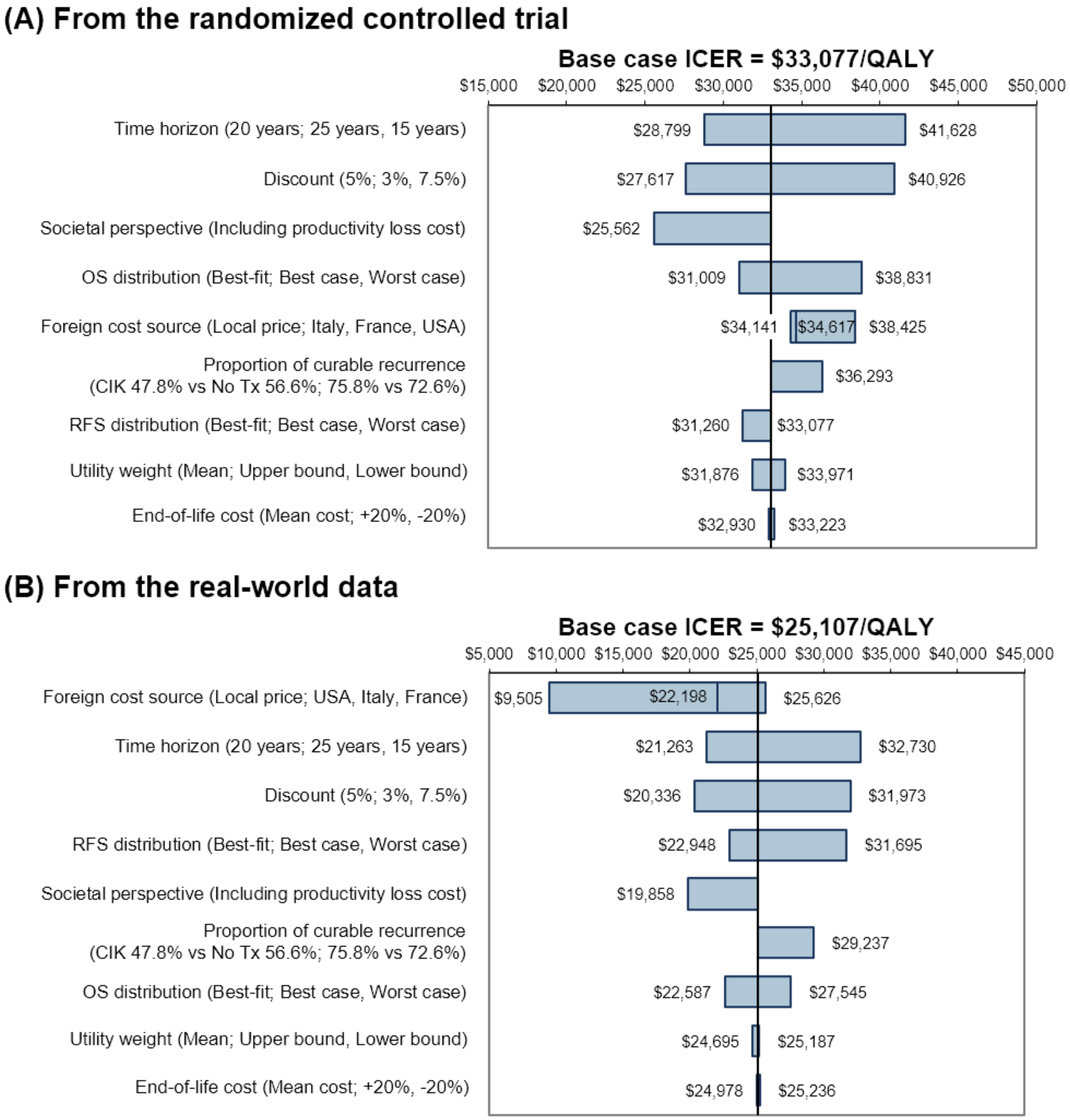

References

1. Lee JH, Lee JH, Lim YS, Yeon JE, Song TJ, Yu SJ, et al. Adjuvant immunotherapy with autologous cytokine-induced killer cells for hepatocellular carcinoma. Gastroenterology. 2015;148(7):1383-91.e6.
2. Lee JH, Lee JH, Lim YS, Yeon JE, Song TJ, Yu SJ, et al. Sustained efficacy of adjuvant immunotherapy with cytokine-induced killer cells for hepatocellular carcinoma: an extended 5-year follow-up. Cancer Immunol Immunother. 2019;68(1):23-32.
3. Yoon JS, Song BG, Lee JH, Lee HY, Kim SW, Chang Y, et al. Adjuvant cytokine-induced killer cell immunotherapy for hepatocellular carcinoma: a propensity score-matched analysis of real-world data. BMC Cancer. 2019;19(1):523.
4. Cadier B, Bulsei J, Nahon P, Seror O, Laurent A, Rosa I, et al. Early detection and curative treatment of hepatocellular carcinoma: A cost-effectiveness analysis in France and in the United States. Hepatology (Baltimore, Md). 2017;65(4):1237-48.
5. Rognoni C, Ciani O, Sommariva S, Tarricone R. Real-World Data for the Evaluation of Transarterial Radioembolization versus Sorafenib in Hepatocellular Carcinoma: A Cost-Effectiveness Analysis. Value Health. 2017;20(3):336-44.
6. Tangka FK, Subramanian S, Sabatino SA, Howard DH, Haber S, Hoover S, et al. End-of-Life Medical Costs of Medicaid Cancer Patients. Health Serv Res. 2015;50(3):690-709.
